# Exploring outdoor workers’ knowledge, attitudes, practices, and perceived risks of heatwaves in Nepal

**DOI:** 10.1371/journal.pone.0329557

**Published:** 2025-08-13

**Authors:** Sudim Sharma, Sabina Marasini, Anjali Joshi, Nripa Raj Dangaura, Lava Raj Timsina, Deepa Guragain, Biraj Man Karmacharya

**Affiliations:** 1 Group for Technical Assistance, Nepal; 2 Health Foundation Nepal, Nepal; 3 Kathmandu University Hospital Dhulikhel Hospital, Nepal; 4 International Drug Development Institute, North Carolina, United States of America; The Chinese University of Hong Kong, HONG KONG

## Abstract

**Background:**

The rise in global temperatures due to climate change has intensified the frequency and severity of heat waves, disproportionately affecting outdoor workers. This is particularly concerning in low- and middle-income countries like Nepal, where inadequate policies and limited awareness leave outdoor workers highly vulnerable. This study explores the knowledge, attitudes, practices (KAP), and perceived risks of heatwaves among outdoor workers in Nepal.

**Methods:**

A cross-sectional mixed-method study was conducted across eight districts in five provinces of Nepal, surveying 356 outdoor workers from five occupational groups: street vendors, agricultural workers, rickshaw drivers/pullers, laborers, and service workers. Eleven focus group discussions (FGDs) were conducted to gain deeper insights. Descriptive statistics were used to assess KAP scores, while Kernel-Based Regularized Least Square (KRLS) analysis examined the variations in practice scores among groups. Thematic analysis was applied to FGDs. The quantitative analysis was done in STATA-14, and the qualitative analysis was conducted manually.

**Results:**

The average age of participants in the study was 37.2 years (SD = 10.5), and just over half (57%) were male. On average, they had worked outdoors for about 10.7 years (SD = 8.6). Among all participants, 43% had heard of heatwaves, 86.2% were aware of heat-related incidents, and 78.6% had personally experienced them. Awareness about heatwave was positively associated with the practices of heat protection for the overall sample (practice score = 1.46, p < 0.001). Age was found to be negatively associated with the practices of heat protection (practice score = −0.03, p < 0.001). Compared to females, males had lesser practices of heat protection (practice score = −0.97, p < 0.001).

**Conclusion:**

Heatwaves pose significant health risks, particularly for vulnerable outdoor workers who often lack knowledge about protective measures. This highlights an urgent need for government-led interventions and awareness programs at both community and policy levels to address and mitigate heat stress.

## Introduction

Climate change has significantly raised global surface temperatures and driven a surge in extreme weather events, particularly heatwaves. As global temperatures continue to rise, heatwaves have become more frequent and intense [1]. These events are characterized by several consecutive days and nights of unusually high temperatures, often accompanied by increased humidity, which poses serious health risks [2]. Prolonged exposure to such conditions can lead to heat stress, manifesting as fatigue, headaches, and more severe heat-related illnesses, such as heatstroke, which can be fatal. Extended exposure to extreme heat can exacerbate existing health conditions and increase the risk of illness and death, making it a critical public health issue [2,3].

Certain populations are particularly vulnerable to the effects of heatwaves. These include individuals with chronic health conditions, outdoor workers, those living in homes with galvanized metallic roofs, and people with lower socioeconomic status [2,4]. The health impacts are significant: between 1998 and 2017, over 166,000 deaths globally were attributed to heatwaves, including more than 70,000 fatalities in Europe alone. Additionally, the number of people exposed to extreme heat increased by nearly 125 million between 2000–2016, highlighting the escalating public health risks associated with heatwaves in a warming climate [5].

Projections indicate that climate-driven increases in frequency, duration, and intensity of heatwaves will continue across South Asia, including Nepal, leading to heightened health risks [6,7]. Under a high-emission scenario (e.g., RCP8.5), the region could experience a substantial rise in the number of heatwave days and average temperature exceeding 35°C [8]. In Nepal, the number of days with wet-bulb temperatures nearing critical physiological thresholds is expected to increase, especially in the Terai region. Estimates suggest that by the 2080s, heat-related mortality in Nepal could reach approximately 53 deaths per 100,000 annually, compared to around 4 per 100,000 during 1961–1990, if no mitigation occurs [9,10]. Heat index projections also indicate a rise in the number of days where apparent temperature exceeds safe limits for outdoor work and physical exertion, further intensifying public health risks. These projections underscore the urgent need for effective public health interventions and climate adaptation strategies tailored to national and regional contexts to mitigate the worsening impact of heatwaves on vulnerable populations. The growing frequency and severity of heatwaves due to climate change presents a major public health challenge, necessitating comprehensive and equity-focused strategies that prioritize vulnerable populations, including outdoor workers, to address both the immediate and long-term effects of extreme heat.

Nepal, with its diverse topography, experiences a wide range of climates from subtropical to alpine. Temperature variations in Nepal are largely shaped by its geography, which spans from the low-lying Terai plains in the south to the high mountainous regions in the north [11]. The Terai plains are Nepal’s hottest areas, where temperatures often exceed 40°C[4,12]. As human bodies struggle to tolerate temperatures above 37°C for extended periods [2], the high temperatures in the Terai make residents especially susceptible to heat-related health hazards. Between 2002 and 2010, Nepal recorded 25 heatwaves, with the highest occurrences in 2009 and 2010. These heat waves resulted in 25 reported deaths and affected 280 individuals [3]. Another study examining the trends of climatic disasters in Nepal revealed that between 1991 and 2021, heatwaves were responsible for 35 deaths [12]. Despite these figures, data on heat-related incidents in Nepal are underreported, largely due to the absence of a systematic reporting mechanism within the healthcare system [3].

The rise in temperature and humidity has also increased outdoor workers’ exposure to humid heat, which can be more dangerous than dry heat. Occupational heat exposure can lead to severe health conditions, including heatstroke [13,14]. Outdoor workers in Nepal frequently perform strenuous manual labor and face heightened health risks due to their work demands in high temperatures. These workers regularly endure temperatures ranging from 36°C to 39°C, exceeding the wet-bulb temperature limit of 35°C, beyond which the human body struggles to regulate heat [15,16]. Combined with the high physical demands of their work, these conditions can significantly elevate the risk of heat stress. Studies indicate that heavy workloads and high temperatures can increase the prevalence of heat-related illnesses by two to three times [17,18].

Awareness and knowledge about heatwaves play a critical role in shaping risk perception and encouraging preventive actions. Informed individuals are more likely to take necessary precautions to protect themselves from heat-related illnesses, while insufficient awareness leaves at-risk groups—such as outdoor workers, the elderly, and people with pre-existing conditions—especially vulnerable [19]. Few studies have explored the knowledge, attitudes, and practices (KAP) related to heatwaves in Nepal, particularly among vulnerable groups. This study aims to fill that gap by assessing outdoor workers’ understanding and perceptions of heatwaves in areas of Nepal that are prone to high temperatures. By evaluating their KAP, this study seeks to identify gaps in awareness and inform strategies to enhance preparedness and resilience among Nepal’s vulnerable populations.

## Methodology

### Study design

A mixed-method study was conducted, combining both quantitative and qualitative approaches to assess outdoor workers’ understanding and risk perception of heatwaves. First, a quantitative assessment using a descriptive cross-sectional design evaluated workers’ knowledge, attitudes, and practices related to heatwaves. This was followed by a qualitative study using thematic analysis of focus group discussions to explore workers’ personal experiences and perceptions in greater depth. During analysis, results were triangulated to enrich interpretation, with qualitative insights explaining quantitative trends, thereby providing a more complete and contextualized understanding. Data collection was conducted over a two-month period from 15 July 2022–28 September 2022.

### Study site

The study was conducted in eight districts across five provinces in Nepal: Kailali (Sudurpaschim province); Rupandehi, Nawalparasi, and Banke (Lumbini province); Morang (Koshi province); Surkhet (Karnali province); Chitwan and Makwanpur (Bagmati province). These districts were selected due to the rising temperature trends observed during the summer months, following the Heat Index classification outlined by the National Weather Service (NWS) [20].

### Study population

We included groups of outdoor workers who are exposed to heat or high temperatures in the selected districts. We targeted the population groups involved in mainly outdoor occupations- street vendors, farmers/agricultural engagement, rikshaw drivers/pullers, labor workers and service workers.

### Sampling technique and sample size

For the qualitative component of the study, purposive sampling was employed, resulting in 11 Focused Group Discussions (FGDs) that included a total of 80 participants. The participants were outdoor workers from various occupations, including agriculture workers/farmers, laborers, rickshaw drivers/pullers, street vendors, and service workers. Each FGD consisted of 6–10 participants. The number of FGDs was determined based on the principle of data saturation [21].

In the quantitative component of the study, a cluster random sampling technique was utilized, enrolling a total of 356 participants primarily engaged in outdoor occupations across eight districts in Nepal. The sample size was guided by findings from the prior studies, which reported a high perception (90%) of heatwaves among the population [3,22]. Using this prevalence estimate (p = 0.90), a margin of error of 5%, and a 95% confidence interval, the initial sample size was calculated using the standard formula for proportions, yielding an estimate of 139 participants. Since cluster sampling was employed, where each cluster was defined as a municipality, the sample size was adjusted for design effect using the formula DEFF = 1 + (m – 1) × ICC, where the average cluster size was 8 and the intra-cluster correlation coefficient was set as 0.20, based on estimates from a Nature Climate Change study [23]. This calculation yielded a design effect of 2.4, which resulted in an adjusted sample size of 334. Accounting for a 5% attrition rate, the final required sample size was set at 351. Ultimately, 356 participants were included in the study. The sampling process involved a total of 44 clusters (municipalities) with 8 participants recruited from each cluster, ensuring both geographic representation and methodological rigor.

### Data collection

For the qualitative component, 11 FGDs were conducted with the study participants using a semi-structured FGD guide. We identified eligible participants and invited those interested at a convenient time and place for the discussions. All FGDs were conducted face-to-face in the Nepali language, following all COVID-19 preventive measures. With the consent of the participants, the discussions were audio recorded.

Similarly, for the quantitative component, a survey was conducted with participants who provided consent. Research assistants administered structured questionnaires through face-to-face interviews. The survey questionnaire consisted of five sections: a) socio-demographic information; b) knowledge related to heatwaves; c) knowledge statements measured on a 5-point Likert scale [ranging from strongly disagree(0) to strongly agree (4)]; d) attitude statements also assessed using 5-point Likert scale [from strongly disagree (0) to strongly agree (4)]; and e) practice related statements with response options of never (0), sometimes (1) and always (2).

Both the questionnaire and the guide were developed based on a literature review and discussions within the research team. Initially prepared in English, they were translated into Nepali for ease of communication. The questionnaire and FGD guides are available upon request from the corresponding author.

#### Study variables.

Data were collected on participants’ age, province, gender, marital status, education level, monthly income, and place of residence (rural or urban). Heatwave knowledge was evaluated using three yes/no questions regarding awareness of heatwaves and related incidents, along with six statements rated on a Likert scale. These responses were recoded for analysis, with one knowledge item reverse coded.

Attitudes toward heatwaves were assessed through six Likert-scale statements, with the responses similarly recoded, and two statements were also reverse coded. Heatwave-related practices were evaluated with thirteen statements rated as “never,” “sometimes,” or “always,” which were recoded to measure the frequency of practices. Summed scores for knowledge (ranging from 0–24), attitudes (0–24), and practices (0–26) were then calculated as continuous variables for further analysis.

### Data analysis

The data analysis was conducted for five occupational groups: street vendors, agricultural workers, rickshaw drivers, laborers, and service workers. For the quantitative analysis, descriptive statistics were used along with the Kernel-Based Regularized Least Square (KRLS) method. KRLS, a nonparametric machine learning method, enables modeling without assuming functional forms, reducing overfitting and the influence of outliers [24] KRLS was chosen over traditional models, such as logistic regression, because it does not require assumptions of linearity or additivity. This capability makes it effective in capturing complex, non-linear relationships and interactions among variables. This flexibility was particularly important given the exploratory nature of the study and the expectation of non-linear associations in workers’ knowledge, attitudes, and risk perception related to heatwaves. Furthermore, KRLS provides interpretable marginal effects, which aid in understanding the influence of individual predictors while maintaining model flexibility [24].

Descriptive frequencies and percentages were calculated, and chi-square tests were conducted to assess statistical significance. To examine associations between knowledge, attitudes, and practice scores, pairwise and Spearman’s rank correlations were utilized, followed by KRLS estimates for practice scores by occupation. All quantitative analyses were performed using Stata 14 with a significance level set at 0.05.

For the qualitative component of the study, FGDs were conducted with participants from each occupational group to explore their experiences, perceptions, and coping strategies related to heatwaves. All FGDs were audio-recorded, transcribed verbatim, and translated into English. The data were analyzed using thematic analysis. A codebook was initially developed based on existing literature (deductive coding) and was subsequently refined by incorporating new codes that emerged from the data (inductive coding). Two researchers independently reviewed the transcripts and applied open coding to identify initial themes. Coding was performed manually using Microsoft Excel. The codes were then compared and refined through discussions and organized into overarching themes using a consensus-driven approach. To ensure analytical rigor, intercoder reliability was assessed by calculating the percentage agreement between coders. Any discrepancies were resolved through iterative discussions until full consensus was achieved.

### Ethical considerations

Ethical approval for the study was granted by the Nepal Health Research Council (ID: 273–2022). All the participants were informed about the study’s objectives and purpose, and written informed consent was obtained from each participant. Additionally, consent was secured to record the discussions during the FGD. The safety and confidentiality of participants were maintained throughout the study.

## Results

The findings are presented in two sections: quantitative findings followed by the qualitative findings. For the quantitative findings, we categorized the study participants into five occupational groups based on their work engagement: street vendor, agriculture, rikshaw drivers, labor workers and service workers.

### Quantitative findings

**Table 1** presents the socio-demographic details of the participants from various occupational groups. The average age of the participants was 37.3 years (SD = 10.6). The sample included a higher proportion of males, representing 57.3% of the participants. A significant majority, 82.87%, were married, while 42.7% had completed only primary education. Additionally, more than half of the participants (64.3%) resided in urban areas.

**Table 1:** Sociodemographic information of the participants.

Variable	In Sample (n = 356)	Street vendor (n = 78)	Agriculture (n = 65)	Rikshaw driver (n = 51)	Labor worker (n = 104)	Service (n = 58)	p-value @
Age (mean ± SD)	37.3 ± 10.6	38 ± 10.9	39.4 ± 10.8	42.4 ± 10.7	37.2 ± 9.5	29.4 ± 6.6	0.0001*
**Province**							
Koshi	55 (15.5)	13 (16.7)	9 (13.9)	1 (2)	24 (23.1)	8 (13.8)	<0.0001
Bagmati	87 (24.4)	19 (24.4)	18 (27.7)	14 (27.4)	19 (18.3)	17 (29.3)	
Lumbini	111 (31.2)	20 (25.6)	17 (26.1)	8 (15.7)	43 (41.3)	23 (39.7)	
Karnali	41 (11.5)	11 (14.1)	6 (9.2)	8 (15.7)	8 (7.7)	8 (13.8)	
Sudurpaschim	62 (17.4)	15 (19.2)	15 (23.1)	20 (39.2)	10 (9.6)	2 (3.4)	
**Gender**							
Male	204 (57.3)	46 (59)	22 (33.9)	47 (92.2)	64 (61.5)	25 (43.1)	<0.001
Female	152 (42.7)	32 (41)	43 (66.1)	4 (7.8)	40 (38.5)	33 (56.9)	
**Marital status**							
Unmarried	61 (17.13)	13 (16.7)	3 (4.6)	8 (15.7)	13 (12.5)	24 (41.4)	<0.001
Married	295 (82.87)	65 (83.3)	62 (95.4)	43 (84.3)	91 (87.5)	34 (58.6)	
**Educational status**							
No formal education	72 (20.2)	16 (20.5)	19 (29.2)	9 (17.7)	24 (23.1)	4 (6.9)	<0.001
Primary education	152 (42.7)	36 (46.2)	28 (43.1)	33 (64.7)	48 (46.1)	7 (12.1)	
Secondary and above	132 (37.1)	26 (33.3)	18 (27.7)	9 (17.6)	32 (30.8)	47 (81)	
**Monthly income (median)**	20000	23500	15000	20000	20000	26000	
**Place of Residence**							
Rural	127 (35.7)	21 (26.9)	51 (78.5)	7 (13.7)	35 (33.7)	13 (22.4)	<0.001
Urban	229 (64.3)	57 (73.1)	14 (21.5)	44 (86.3)	69 (66.3)	45 (77.6)	

*Kruskal-Wallis test for significance;@ Chi-square test

**Table 2** highlights how participants’ awareness, experiences, and Knowledge, Attitude, and Practice (KAP) scores related to heatwaves vary depending upon their occupation. Among the total participants, only 43% of the participants had heard about a heatwave. But when asked if they had heard about heat-related incidents (like heat exhaustion, heat rash, nausea, dizziness due to exposure to heat, etc.), 86.2% of participants gave a positive response. And 78.6% of the participants responded that they had experienced the heat incidents themselves. These findings show that though the participants had experienced the effect of a heatwave, they were not aware of the term itself. The average knowledge score was 12.6 ± 4.9, and the practice score was 14.5 ± 3.9. Among the occupation groups, the service workers were found to be comparatively more aware about heatwaves as a higher proportion of them had heard about heatwaves, heat-related incidents, and had higher knowledge and practice scores. 

**Table 2:** Heatwave related information of participants by their occupation.

Variable	In Sample (n = 356)	Street vendor (n = 78)	Agriculture (n = 65)	Rikshaw driver (n = 51)	Labor worker (n = 104)	Service (n = 58)	p-value@
**Heard about heatwave**							
No	203 (57)	54 (69.2)	39 (60)	41 (80.4)	62 (59.6)	7 (12.1)	<0.0001
Yes	153 (43)	24 (30.8)	26 (40)	10 (19.6)	42 (40.4)	51 (87.9)	
**Heard about heat related incidents**							
No	49 (13.8)	15 (19.2)	5 (7.7)	8 (15.7)	19 (18.3)	2 (3.5)	0.025
Yes	307 (86.2)	63 (80.8)	60 (92.3)	43 (84.3)	85 (81.7)	56 (96.5)	
**Experienced heat incidents**							
No	76 (21.4)	19 (24.4)	12 (18.5)	6 (11.7)	26 (25)	13 (22.4)	0.355
Yes	280 (78.6)	59 (75.6)	53 (81.5)	45 (88.2)	78 (75)	45 (77.6)	
**KAP Score: mean **± SD							
Knowledge score (0–24)	12.6 ± 4.9	11.9 ± 5.2	13.2 ± 5	10.4 ± 4.9	12.9 ± 4	14 ± 4.9	0.0010*
Attitude score (0–24)	12.5 ± 4.3	12.3 ± 4.7	11.7 ± 4.1	11.8 ± 4.4	12.7 ± 3.7	13.6 ± 4.8	0.1646
Practice score (0–26)	14.5 ± 3.9	14.2 ± 4.6	15 ± 3.7	12 ± 3.3	14.4 ± 3.3	16.9 ± 3.6	0.0001*

*Kruskal Wallis test for significance; @ Chi-square test

**Table 3** shows KRLS estimates of practice scores across occupations, highlighting several key influences. Knowledge scores positively impacted practice scores in all groups except for street vendors. The provincial distribution shows that practice scores were lower in Karnali (practice scores = 0.89, p = 0.007) and higher in Koshi (practice scores = 1.58, p = 0.000) compared to Bagmati. In Karnali province, except rickshaw drivers, all other occupational groups had less practices of heat protection. Likewise, in Koshi province, except for service workers, other occupation groups had better practices of heat protection. Awareness about heatwave was positively associated with the practices of heat protection for the overall sample (practice score = 1.46, p < 0.001). Age was found to be negatively associated with the practices of heat protection (practice score = −0.03, p < 0.001). Compared to females, males had lesser practices of heat protection (practice score = −0.97, p < 0.001), and the least practices were observed among the male service workers (practice score = −2.1, p < 0.001). In education, compared to the no formal education group, primary education was associated with slightly better practice scores (practice score = 0.76, p = 0.003). Among occupational groups, rikshaw drivers having primary education (practice score = 1.62, p < 0.001) and agriculture workers with secondary and above educational level (practice score = 1.01, p < 0.001) had comparatively better practices of heat protection. 

**Table 3:** Multivariable Kernel based regularized least square average estimate of overall practice score: average (p-value).

Variable	In Sample (n = 356)	Street vendor (n = 78)	Agriculture (n = 65)	Rikshaw driver (n = 51)	Labor worker (n = 104)	Service (n = 58)
Attitude Score	0.03 (0.893)	0.13 (0.003)	0.003 (0.911)	0.13 (<0.001)	−0.02 (0.379)	−0.03 (0.356)
Knowledge score	0.21 (<0.001)	0.02 (0.401)	0.15 (<0.001)	0.06 (0.003)	0.1 (<0.001)	0.1 (0.001)
Knowledge-attitude interaction	0.0009 (0.238)	0.001 (0.231)	0.005 (<0.001)	0.006 (<0.001)	0.0007 (0.460)	−0.006 (<0.001)
**Province**						
Bagmati	Ref.					
Koshi	1.58 (<0.001)	1.92 (<0.001)	1.26 (<0.001)	0.93 (0.004)	1.39(<0.001)	−0.04 (0.925)
Lumbini	0.44 (0.094)	0.65 (0.078)	−0.05 (0.809)	−0.57 (0.033)	1.28 (<0.001)	−0.23 (0.648)
Karnali	−0.89 (0.007)	−2.53 (<0.001)	−1.75 (<0.001)	1.72 (<0.001)	−1.47 (<0.001)	−2.37 (<0.001)
Sudurpaschim	−0.19 (0.484)	−0.12 (0.739)	−0.09 (0.740)	1.1 (<0.001)	−0.14 (0.637)	0.12 (0.648)
**Heard about heatwave**						
No	Ref.					
Yes	1.46 (<0.001)	0.67(0.101)	0.61 (0.043)	1.13 (<0.001)	0.57 (0.038)	0.82 (0.056)
**Age**	−0.03 (<0.001)	−0.05 (<0.001)	−0.009 (0.319)	−0.02 (0.002)	0.01 (0.275)	−0.09 (0.003)
**Gender**						
Female	Ref.					
Male	−0.97 (<0.001)	0.61 (0.105)	−0.91(0.004)	1.09 (<0.001)	−0.89 (<0.001)	−2.1 (<0.001)
**Education level**						
No formal education	Ref.					
Primary education	0.76 (0.003)	−0.73 (0.057)	0.72 (0.016)	1.62 (<0.001)	0.55 (0.036)	0.36 (0.402)
Secondary and above	0.41 (0.163)	0.64 (0.110)	1.01 (<0.001)	0.64 (0.004)	0.24 (0.371)	−0.24 (0.651)

Note: results are presented as average score (p-value).

### Qualitative findings

The findings from 11 FGDs involving a total of 80 participants, are organized and presented under five distinct thematic areas:

#### Theme 1: Knowledge of heatwave.

Most participants recognized that increase in temperature, as well as colder winters, are signs of climate change. They noted experiencing shifts in seasonal patterns with summers becoming extremely hot and winters turning excessively cold. According to the participants, deforestation and urbanization are the primary contributors to rising temperatures.

*“Because of climate change, the sun is getting hotter, winters are exceptionally cold, and the rainy season is becoming shorter.”*

When asked about temperature differences during the summer over the last 10–12 years, everyone agreed that the heat has significantly increased compared to the past. However, most participants were unfamiliar with the term “heatwave” (known as “Luu” in Nepali). When alternative synonyms were offered, they acknowledged having heard of it. Many participants admitted to experiencing heatwaves, which they said are most common during the months of Falgun to Jestha (February to June).

*“People from the older generation often talk about heatwaves (Luu). The occurrence typically starts in Falgun (February) and happens more frequently between Falgun and Jestha (February to June). Nowadays, it seems like summers are lasting longer.”*

#### Theme 2: Perceptions regarding the health and environmental impacts of heatwave.

Participants reported that heatwaves significantly impact both health and the environment. In terms of health, the most common problems experienced during heatwaves include the common cold, fever, rashes, headache, nausea and loss of appetite. Some participants noted that diseases such as pneumonia, dengue, typhoid tend to be more prevalent on hotter days. They expressed concerns about having to work outdoors during the day without adequate measures to manage high temperatures, which negatively affects their health.

Participants identified several major factors contributing to the increased risk of heatwaves, including industrialization, population growth, unplanned urbanization and deforestation. The groups most affected by these heatwaves include children, outdoor workers, and the elderly. The environmental impacts of heatwaves are also significant, particularly in agriculture. Many participants observed that crops are turning red and drying out due to drought conditions. They reported challenges in agriculture, such as dryness and difficulties with irrigation due to lack of rainfall.

*“The crops are turning red due to the heat. Last year, there was no rainfall during the rainy season, and it heavily rained during the harvest time. This year, we are seeing the same trend. The seasons seem to be changing over time.”*

*“The elderly people and school going children are affected more due to heat. Along with them the people who work in outdoor setting are also affected more due to increasing heat.”*

#### Theme 3: Protective measures.

Participants reported that heatwaves and prolonged exposure to the extreme sun are harmful. However, they also indicated that they often have no choice but to work outside, even in high temperatures.

*“We have no option but to be exposed to the sun at work. This job is our only means of supporting our daily lives. If we do not work during the day, we will not have food in the evening.”*

Most participants mentioned that they attempted to practice some protective behaviors while working in the sun but they felt that these measures were often not practical. Common protective strategies included taking periodic breaks in the shade, drinking plenty of water, wearing hats or shawls to protect their heads, and wearing sunglasses (especially among male participants) and so on.

#### Theme 4: Role of government and concerned authorities in minimizing the impact/effect of heatwave and risk communication.

Participants made several major recommendations for addressing the impact of heatwaves, including afforestation, preserving natural resources, raising awareness about preventive measures, and revising relevant policies etc.

One participant pointed out, *“There are laborers who work outside. If policies were made to allow work during the morning from 6 am for 7 hours, like in some foreign countries, it would make things easier for us. However, in our country, if we don’t work all day, we don’t have anything to eat at night.”*

Many participants were not well-informed about how to prevent the health impacts associated with heatwaves. They mentioned that working in hot temperature had become a habit for them. The majority said they had not received any information or updates regarding heatwaves from organizations or the government. Those who had heard the term ‘heatwave’ primarily learned it from family members and relatives.

Some participants recalled hearing news on the radio about rising temperature, but they had limited knowledge of heatwave prevention measures. They expressed uncertainty about whether heatwaves are a serious concern due to their lack of comprehensive understanding of the issue.

One participant stated, “*We have experienced an increase in temperature, but we’re unaware of its potential consequences. Without understanding the effects, we can’t confidently assess whether heatwaves are a serious issue.”*

Most participants admitted they had never heard about heatwaves from any news sources.

#### Theme 5: Policy/Organizational support.

The participants expressed that they do not have any expectations of support from the policy or organizational levels. They conveyed a sense of disillusionment regarding higher authorities. Many participants were unaware of how serious the heatwave issue is, as they lacked knowledge about its consequences and impacts. This ignorance may contribute to their reluctance to seek support. Additionally, participants mentioned that the government has not met their expectations regarding basic needs, such as access to water, health services, education and employment. As a result, most expressed little anticipation for assistance.

*“We have been struggling with the local government to meet our basic needs, like a reliable drinking water supply. The government has not provided us with relief materials during floods or other disasters. In this context, we do not expect any support from the government regarding heatwaves or similar issues.”*

## Discussion

This study was conducted to assess the existing knowledge, attitudes, practices (KAP), and risk perception related to heatwaves among outdoor workers in Nepal. The results revealed a significant lack of awareness regarding heatwaves, with only 47% of participants reporting any prior knowledge of the phenomenon. The finding was corroborated by qualitative interviews, during which participants frequently described symptoms such as headaches, dizziness, and nausea associated with extreme heat but did not explicitly link these symptoms to the broader climatic phenomenon of heatwaves. This indicates a substantial knowledge gap among outdoor workers, a group particularly vulnerable to the adverse effects of heatwaves. This lack of awareness is consistent with previous studies conducted in Greece, Bangladesh, Germany, and China, which also observed limited heatwave awareness among vulnerable populations, such as outdoor workers and general public [25–28].

Despite many participants having experienced heat-related incidents, their understanding of the term “heatwave” was limited. This suggests that while outdoor workers may recognize symptoms and consequences of extreme heat, they lack awareness of the broader condition known as a “heatwave.” Addressing this knowledge gap is crucial for enhancing preparedness and mitigating health risks associated with extreme heat.

The study also highlighted significant disparities in KAP levels among different groups of outdoor workers. Knowledge and practices were found to be lowest among rickshaw drivers, while attitudes were notably lower among agricultural workers. In contrast, service workers demonstrated the highest levels of KAP, indicating a better understanding and implementation of practices to mitigate heatwave risks.

Various attributes such as age, sex, and education were significantly associated with the different practices among outdoor worker groups (Table 3). Workers aged below 35 years had significantly higher KAP scores compared to older age groups (p < 0.05). Qualitative insights supported this finding, showing that younger respondents appeared more engaged with digital media and more receptive to public health messaging. This aligns with international evidence indicating that younger populations are generally more adaptable to climate-related health risks [29], as found in a study conducted in China, where younger individuals were more receptive to climate health warnings [28]. These findings underscore the importance of targeted educational interventions that consider these demographic factors to effectively raise awareness and improve heatwave preparedness among all outdoor workers.

This study indicates that there is a need to improve outdoor workers’ knowledge and attitudes towards heatwaves, which is crucial for promoting safer practices and reducing health risks. Public health initiatives should prioritize comprehensive education and training programs tailored to meet the specific needs of various worker groups. Additionally, policies should enhance the dissemination of information on heatwave preparedness and response, ensuring that all outdoor workers are equipped with the necessary knowledge and tools to protect themselves from the dangers of extreme heat.

While participants generally recognized rising temperatures, they attributed these changes to factors such as deforestation, rapid urbanization, and land-use changes. This reflects some awareness of the drivers of climate change but indicates a limited understanding of potential mitigation or adaptation strategies.

According to the National Adaptation Programme of Action (NAPA) 2010, Nepal is expected to experience temperature increases of 1.4 °C by 2030 and up to 4.7 °C by 2090, leading to more hot days and nights [30]. However, participants were uncertain about effective mitigation strategies, highlighting gaps in community-level communication regarding climate action. Their experiences with increasing temperatures and extreme weather align with projected models, underscoring the urgency for localized climate adaptation planning, risk communication, and fostering conditions for climate-resilient development [31].

Field observations and interviews with participants revealed that most workers were exposed to peak heat hours without adequate rest or shift adjustment policies. Working during peak summer months without sufficient protection or adaptation measures significantly impacts the health and productivity of outdoor workers, leading to heat stress. In many cases in Nepal, there is no structured policy or provision to facilitate work under extreme heat conditions. While some outdoor workers reported using protective measures such as drinking water, wearing caps, and avoiding prolonged sun exposure, these practices were inconsistently followed. Daily wage earners, in particular, struggled to take breaks or modify work hours due to economic constraints. Passive heat avoidance strategies, such as seeking shade, were also largely impractical in many outdoor settings [32,33]. These findings are consistent with studies conducted in Asia, which show that informal workers often lack the institutional and occupational support needed to effectively manage and mitigate heat stress [34].

Considering the health impact of heatwaves, elderly individuals, women, children and outdoor workers are identified as the most vulnerable groups [35,36]. Poor and low-income workers are especially at risk, as the daily wages often serve as their sole income source to support their families. A study conducted in western Nepal indicates that such workers could lose up to 23% of their income if they reduce working hours during a heatwave [4] – a trade-off only a few can afford. This highlights the economic dimension of heatwave vulnerability, especially in low-income groups.

Participants described symptoms such as dizziness, nausea, fatigue, and headaches, which are well-documented signs of heat stress and consistent with findings from previous studies [37,38]. Despite experiencing these symptoms, many participants had a low perception of personal risk, a phenomenon also noted in the literature. People often underestimate their risk even when they belong to a high-risk group [39,40]. Similar to our findings, other studies report that knowledge about heatwave protective measures and practices is significantly associated with personal characteristics such as gender, age, and educational level [39,41]. Although increased knowledge was positively linked to better practices, the translation of this knowledge into action was inconsistent. Some participants admitted that even when they understood the importance of staying hydrated, they often failed to act on it due to workplace constraints. This confirms observations from a study conducted in Saudi Arabia [42]. Therefore, behavior change communication (BCC) is essential and must be targeted toward vulnerable groups [43].

The silent nature of heat, which causes no visual destruction, results in a low prioritization of risk by government bodies, even as mortality risks continue to rise. The full extent of heat’s impact in Nepal remains largely unknown, as heat-related illnesses and excess deaths are not systematically tracked. Additionally, there is a lack of studies investigating heat trends, mortality, and morbidity across different populations [44]. As temperatures are projected to rise across the country, heat-related morbidity and mortality will increasingly become a concern in Nepal. Under a high emissions scenario, heat-related deaths are expected to increase from a baseline of four deaths per 100,000 people annually to 53 deaths per 100,000 by 2080 [9]. This underscores the urgent need for early warning systems and response plans as a critical public health issue.

Effective risk communication has been identified as a pivotal factor in reducing mortality during heatwaves. This includes the nature of risk messages, how recipients perceive and respond to them, and how effectively authorities communicate with the general public [45]. Both quantitative and qualitative findings reveal a lack of accessible and trusted channels for disseminating heatwave warnings. Heatwaves not only affect physical health but also have significant implications for mental well-being, contributing to conditions such as anxiety, depression and aggression, with varying effects across different community groups [46,47]. Therefore strategies for mitigating heatwaves and heat stress must be communicated in a targeted and tailored manner, particularly addressing the needs of the most vulnerable populations, as generic or blanket warnings risk being overlooked [48].

Despite our best efforts, it is important to interpret the findings of this study in light of certain limitations. For instance, the results might not be nationally representative, as we have analyzed a small sample of data from selected study sites. However, this research provides insights into heat stress perception among a vulnerable group- outdoor workers. While the workers’ self-assessments may be subjective or misinterpreted, most responses are consistent with the previously identified impacts of heat stress. Furthermore, since the study is cross-sectional, establishing a cause-and-effect relationship is not possible.

## Conclusion

Climate change-related disasters, such as heatwaves, have numerous adverse effects on human health, social capital, and the entire ecosystem. In a country like Nepal, heatwaves can be particularly detrimental to vulnerable populations. With the average maximum temperature during the peak summer months in the Terai region exceeding 39^o^ Celsius, the risk of heat stress and associated health complications is significantly heightened. Prolonged exposure to high temperatures has severely impacted outdoor workers, who often lack adequate knowledge, hold misconceptions about heatwaves, and rarely utilize protective measures. Furthermore, there has been little or no effort by relevant agencies to address heat stress in both indoor and outdoor working environments. The coping strategies currently employed by workers are inadequate to mitigate the health risks posed by extreme heat. Raising awareness among working groups and the wider community is critical. Therefore, it is strongly recommended that the Government of Nepal urgently prioritizes the development and implementation of a comprehensive national heat action plan – one that assesses social vulnerabilities, promotes cost-effective interventions, and co-designs inclusive climate policies aimed at protecting the health and livelihood of at-risk populations.

## Supporting information

S1 Data Quantitative dataset. (XLSX)
